# Computer-assisted navigation in Birmingham hip resurfacing: A case report

**DOI:** 10.1177/2050313X18819641

**Published:** 2018-12-21

**Authors:** Ritesh Shah, Jessica R Benson, Jeffrey M Muir

**Affiliations:** 1 Department of Orthopedic Surgery, Illinois Bone & Joint Institute, Morton Grove, IL, USA; 2 Department of Orthopedic Surgery, Advocate Lutheran General Hospital, Park Ridge, IL, USA; 3 Department of Orthopedic Surgery, NorthShore University HealthSystem – Skokie Hospital, Skokie, IL, USA; 4 Intellijoint Surgical, Waterloo, ON, Canada

**Keywords:** Orthopaedics/rehabilitation/occupational therapy, surgery, Birmingham hip resurfacing, computer-assisted navigation

## Abstract

Component malpositioning during Birmingham hip resurfacing increases the risk for component wear, metallosis, component loosening, and the likelihood of dislocation and revision surgery. Computer-assisted navigation can increase the accuracy to which components are placed, and the utilization of this technology in Birmingham hip resurfacing is increasing. The present report summarizes the accuracy of acetabular component positioning in a Birmingham hip resurfacing case utilizing navigation. Intraoperative C-arm fluoroscopy following the use of the navigation tool confirmed excellent seating, positioning, and stability of the acetabular component. In addition, post-operative antero-posterior radiographs confirmed device accuracy and revealed a stable joint with no evidence of acetabular loosening or femoral fracture. Computer-assisted navigation may therefore be an effective tool to improve the accuracy of component positioning during Birmingham hip resurfacing.

## Introduction

Hip resurfacing is a bone-sparing alternative to total hip replacement (THA) that accounts for approximately 1% of hip arthroplasties in the United States.^1^ The Birmingham hip resurfacing (BHR) system (Smith & Nephew, Andover, MA, USA) is a US Food and Drug Administration (FDA)-approved metal-on-metal (MoM) implant, effective in providing joint stability and longevity to patients seeking less invasive surgical treatment for debilitating hip disease.^2,3^ As with THA, however, component malpositioning can lead to detrimental post-operative outcomes and is a leading cause of dislocation and revision surgery.^1^ Computer-assisted navigation may help to reduce the risk of component malpositioning in such cases by improving the accuracy with which components are placed intraoperatively. Indeed, a growing body of literature shows promising results for the use of computer-assisted navigation in both THA^4^ and hip resurfacing procedures.^5–12^ While resurfacing studies have largely evaluated the accuracy of computer-assisted navigation for femoral component placement,^6–8,11,13^ the accuracy of computer-assisted navigation associated with acetabular component positioning is less characterized.^10,12^ The present report summarizes the use of an emerging, imageless navigation tool in a case of BHR, where navigation accurately measured the acetabular component position intraoperatively.

## Case report

### Patient presentation

A 48-year-old male presented with a chief complaint of bilateral hip pain, more prominent on the right side. The pain was described as intermittent, but significantly progressing in the most recent year, with daily occurrence. The pain was constant and worsened when walking, during prolonged periods of sitting, with sitting to standing, and with physical exercises including running. The patient noted a severe limitation in mobility, experienced with simple activities such as putting on socks and shoes. Past medical, family, and social histories were unremarkable. Conservative management including anti-inflammatory medication, activity modification, icing, home exercising, stretching, and resting had not provided significant relief.

### Orthopaedic examination and diagnosis

Initial orthopaedic examination of the right hip revealed a range of motion of 0° to 90° of flexion with pain at end range, internal rotation (IR) in flexion 5°, external rotation (ER) in flexion 40°, abduction 40°, and adduction 10°. Anterior impingement test on the right side and Patrick’s test to the groin were both positive. On examination of the left hip, range of motion was 0° to 95°, IR in flexion 10°, ER in flexion 50°, abduction 50°, and adduction 10°. Anterior impingement testing on the left was positive. Abductor strength was 5/5 bilaterally. No deformities were identified, and neurological status was intact.

**Figure 1.**
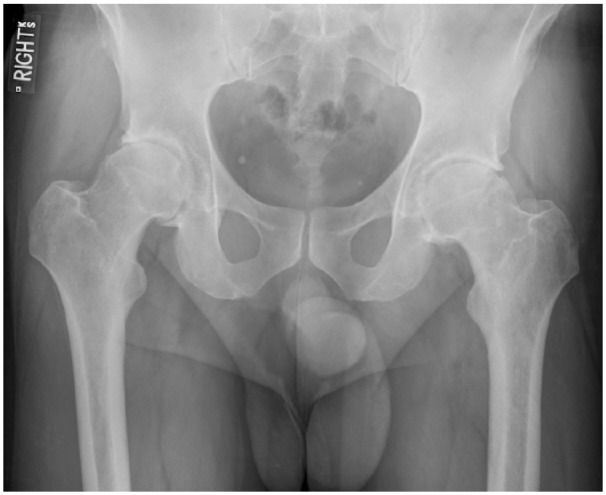
Pre-operative radiograph: pre-operative AP plain film radiograph depicting bilateral hip osteoarthritis with the presence of osteophytes, joint space narrowing, sclerosis, and cam-type femoroacetabular impingement.

Plain film radiographs revealed bilateral hip osteoarthritis with the presence of osteophytes, joint space narrowing, sclerosis, and cam-type femoroacetabular impingement (Figure 1). Based on patient history, age, and examination findings, final diagnosis was bilateral hip osteoarthritis, right hip greater than the left. Treatment options for the right hip included cortisone injection, THA, or BHR. After discussing the risks and benefits of each procedure, the patient opted for right BHR due to his active lifestyle.

### Treatment

**Figure 2.**
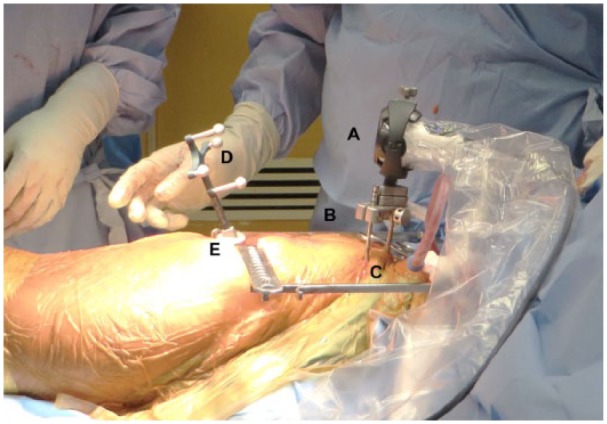
The Intellijoint HIP^®^ navigation camera (A) is enclosed in a sterile drape and magnetically attaches to the pelvic platform (B). The pelvic platform is installed on the ipsilateral iliac crest using two pelvic screws (C). The tracker (D) is magnetically attached to the femoral platform (E). The camera captures movements of the tracker and relays the information to a workstation for review by the surgeon.

Surgery was performed with the assistance of C-arm fluoroscopy and Intellijoint HIP^®^ (Intellijoint Surgical Inc., Waterloo, ON, Canada; off-label use), a 3D mini-navigation tool currently approved for use in posterior, lateral, and direct anterior approaches for THA. While this device has received clearance from the FDA for use in primary and revision THA, it has not been evaluated for use in BHR. The posterior application of the navigation device was followed, which has been described in detail previously^14^ (Figure 2).

### Surgical technique

The patient was placed right side up in the lateral decubitus position and stabilized using a pegboard. The right hip and lower extremity were prepped and draped in usual sterile fashion, followed by a confirmed surgical time out. Two 5-mm stab incisions were made at the iliac crest to accommodate the pelvic screws, pelvic platform, and camera of the navigation system, at which point the horizontal and frontal planes of the patient were registered. A 12-cm posterolateral incision was made and the tensor fascia latae and gluteus maximus fascia were incised. The sciatic nerve was palpated and protected. The gluteus medius and minimus were also protected. Short external rotators and quadratus femoris tissue were incised, leaving a cuff to prevent medial femoral circumflex bleeding. Hemostasis was adequate throughout the entire procedure and the sciatic nerve was palpated and protected throughout the entire procedure. A femoral disc was placed on the lesser trochanter to accommodate the tracker of the navigation tool. Hip biomechanics were registered including baseline leg length. Subsequently, the posterior capsule was incised in a U-shaped capsulotomy. The hip was dislocated atraumatically. Circumferential release of the capsule was performed. With the assistance of C-arm fluoroscopy, a guide pin was placed at the centre of the femoral neck at an angle of approximately 138°. C-arm fluoroscopy and biplanar imaging demonstrated excellent positioning of the guide. The femoral head was prepared per usual fashion using barrel reamer, chamfer reamer, and spherical reamer to create a spherical femoral head. At this point, a femoral cup trial was placed. Excellent fixation was noted without any notching or impingement. Next, the femur was transitioned anteriorly using assistance and blunt retractors. The acetabulum was visualized circumferentially with difficulty due to the stiffness of the hip. The labrum and pulvinar were excised and medial wall was visualized. Sequential reaming was performed. Medialization was excellent without a breach, with great cortical cancellous bleeding bed, and with sequential reamers in 1- and 2-mm increments. Next, a 58-mm acetabular component was impacted in place using the navigation system to confirm excellent angulation at 44° inclination and 20° anteversion, noting excellent seating, alignment, and stability. C-arm fluoroscopy demonstrated excellent positioning of the acetabular component with grade medialization and adequate seating. Next, Simplex cement was mixed per usual fashion and placed in the femoral head which was then impacted into place. Excellent fixation was noted. Cement was allowed to harden, with excess cement removed. Hip was relocated atraumatically. Hip range of motion and stability tested excellent. The navigation system was utilized to confirm baseline leg length restoration, followed by the removal of all navigation-related materials. Copious lavage was performed followed by closure. Skin glue was applied. Aquacel dressing was placed. Patient was awakened, extubated, and brought back to the recovery room in stable condition with no complications noted.

### Follow-up

**Figure 3.**
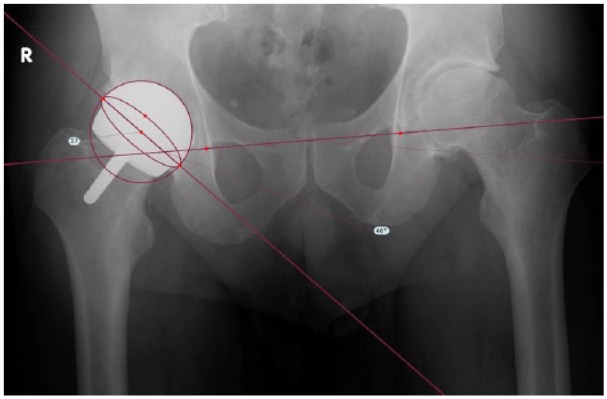
Post-operative radiograph: a 12-week, AP pelvis radiograph is depicted, showing BHR that is well aligned with no evidence of loosening and no femoral neck fracture. TraumaCad overlay (Brainlab, Chicago, IL, USA) depicts post-operative cup position measured at 46° inclination and 17° anteversion.

Post-operatively, the patient was doing extremely well with no pain and full return of mobility. At his 12-week follow-up visit, the patient stated he was back to most of his physical activities and was happy with his progress. An antero-posterior (AP) pelvis x-ray of the right hip showed BHR that was well aligned with no evidence of loosening and no femoral neck fracture (Figure 3).

## Discussion

BHR is considered safe and stable, as many cases describe strong mid- to long-term results, including 88%–99% joint survivorship reported at ⩾5 years.^15,16^ Commonly performed in younger, active, adult males, BHR conserves the femoral head and can produce excellent functional scores post-operatively.^3,17^ However, incorrect positioning of components during BHR can lead to early complications and early BHR failure.^18,19^ While inaccuracies in positioning related to the femoral component increase the risk of loosening and fracture,^20,21^ acetabular component malposition is associated with accelerated wear, impingement, and early dislocation.^22^ The utilization of computer-assisted navigation during resurfacing procedures has largely focused on the positioning of the femoral component.^6–8,11,13^ In the present report, the accuracy of a navigation tool for acetabular measurements (anteversion and inclination) and leg length during BHR was evaluated.

Following patient registration and hip exposure, device measurements of intraoperative cup position were relayed in real time and allowed for a final intraoperative position of 44° inclination and 20° anteversion, selected by the operating surgeon (R.S.). Following navigation, excellent seating, alignment, and stability of the acetabular component were confirmed using C-arm fluoroscopy. Post-operatively, radiographic analysis (TraumaCad; Brainlab) measured a final cup position of 46° inclination and 17° anteversion, indicating device accuracy to within 2° and 3° of radiographic values (Figure 3). The device was also utilized intraoperatively to monitor leg length and ensured restoration following hip relocation. This was confirmed radiographically, as a 1-mm difference in leg length was observed between pre- and post-operative radiographic measurements.

These results are consistent with previous reports confirming the accuracy of computer-assisted navigation in resurfacing.^10,12^ A recent study by Vigdorchik et al.^12^ utilizing the same navigation tool reported device accuracy to within 0.7° and 3° of post-operative radiographic values and restored a pre-operative 3-mm leg length discrepancy to 0 mm following the procedure. In turn, Romanowski and Swank^10^ assessed inclination accuracy following the use of intraoperative navigation and reported no significant difference between pre- and post-operative inclination measurements. The ability of computer-assisted navigation to accurately match target values of cup position is critical for reducing the risk of adverse patient outcomes, such as accelerated component wear resulting from steep inclination angles.^23,24^ The present report showcased the ability of computer-assisted navigation to accurately measure cup position intraoperatively. Adverse outcomes may be preventable with technologies that promote accuracy and reduce the risk of component wear resulting from component malposition.

Limitations of this study first include the inability of the navigation tool to assist with guidance of the femoral pin and cap. This restricted navigation assistance to the acetabular component only. However, this was accommodated for with the use of C-arm fluoroscopy to visualize femoral component position. In turn, the device was able to track leg length changes throughout surgery and accurately restored leg length to within 1° of the radiographic pre-operative position. This is of value as leg length discrepancies are now a leading cause for litigation against orthopaedic surgeons following THA.^25^ The second major limitation of these findings is the singular case examined. The use of the navigation tool in the present report shows promising results for acetabular component navigation during BHR, but further clinical evidence is required. While the device was able to accurately measure anteversion and inclination intraoperatively, these findings should be tested in a larger sample size.

## Conclusion

The present case reported a BHR procedure, wherein the utilization of an imageless navigation tool allowed for accurate component positioning and leg length restoration. These findings are congruent with previous reports on the utilization of computer-assisted navigation during hip resurfacing procedures and provide further insight for the benefits of navigation in BHR specifically.

## References

[1] American Joint Replacement Registry. Fourth AJRR Annual Report on Hip and Knee Arthroplasty Data, 2017, http://www.ajrr.net/media-news/press-releases/500-ajrr-releases-2017-annual-report-on-hip-and-knee-arthroplasty-data

[2] McMinn D Treacy R Lin K et al Metal on metal surface replacement of the hip. Experience of the McMinn prosthesis. Clin Orthop Relat Res 1996; 329S: S89–S98. 10.1097/00003086-199608001-00009 8769326

[3] Ortiz-Declet VR Iacobelli DA Yuen LC et al Birmingham hip resurfacing vs total hip arthroplasty: a matched-pair comparison of clinical outcomes. J Arthroplasty 2017; 32(12): 3647–3651. 28711342 10.1016/j.arth.2017.06.030

[4] Xu K Li YM Zhang HF et al Computer navigation in total hip arthroplasty: a meta-analysis of randomized controlled trials. Int J Surg 2014; 12(5): 528–533. 24583365 10.1016/j.ijsu.2014.02.014

[5] Hess T Gampe T Kottgen C et al [Intraoperative navigation for hip resurfacing. Methods and first results]. Orthopade 2004; 33(10): 1183–1193. 15316600 10.1007/s00132-004-0693-5

[6] El Hachmi M Penasse M Our midterm results of the birmingham hip resurfacing with and without navigation. J Arthroplasty 2014; 29: 808–812. 24140277 10.1016/j.arth.2013.09.014

[7] Bailey C Gul R Falworth M et al Component alignment in hip resurfacing using computer navigation. Clin Orthop Relat Res 2009; 467: 917–922. 18972176 10.1007/s11999-008-0584-x PMC2650050

[8] Hodgson A Helmy N Masri BA et al Comparative repeatability of guide-pin axis positioning in computer-assisted and manual femoral head resurfacing arthroplasty. Proc IMechE, Part H: J Engineering in Medicine 2007; 221(7): 713–724. 10.1243/09544119JEIM284 18019459

[9] Cobb JP Kannan V Brust K et al Navigation reduces the learning curve in resurfacing total hip arthroplasty. Clin Orthop Relat Res 2007; 463: 90–97. 17603387 10.1097/BLO.0b013e318126c0a5

[10] Romanowski JR Swank ML. Imageless navigation in hip resurfacing: avoiding component malposition during the surgeon learning curve. J Bone Joint Surg Am 2008; 90(Suppl. 3): 65–70. 18676939 10.2106/JBJS.H.00462

[11] Seyler TM Lai LP Sprinkle DI et al Does computer-assisted surgery improve accuracy and decrease the learning curve in hip resurfacing? A radiographic analysis. J Bone Joint Surg Am 2008; 90(Suppl. 3): 71–80. 18676940 10.2106/JBJS.H.00697

[12] Vigdorchik JM Elbuluk A Benson JR et al Birmingham hip resurfacing using a novel mini-navigation system: a case report. J Orthop Case Rep 2018; 8(1): 48–52. 29854693 10.13107/jocr.2250-0685.994 PMC5974677

[13] Mann SM Kunz M Ellis RE et al Component position and metal ion levels in computer-navigated hip resurfacing arthroplasty. J Arthroplasty 2017; 32: 119–124. 27430186 10.1016/j.arth.2016.06.028

[14] Paprosky WG Muir JM. Intellijoint HIP^®^: a 3D mini-optical navigation tool for improving intraoperative accuracy during total hip arthroplasty. Med Devices Evid Res 2016; 9: 401–408. 10.2147/MDER.S119161 PMC5125765 27920583

[15] Halawi MJ Brigati D Messner W et al Birmingham hip resurfacing in patients 55 years or younger: risk factors for poor midterm outcomes. J Arthroplasty 2017; 32(6): 1880–1883. 28108170 10.1016/j.arth.2016.12.044

[16] Frew N Johnson G. Survival of the Birmingham hip resurfacing in young men up to 13 years post-operatively. Acta Orthop Belg 2017; 83(1): 67–73. 29322897

[17] Matharu GS McBryde CW Pynsent WB et al The outcome of the Birmingham Hip Resurfacing in patients aged <50 years up to 14 years post-operatively. Bone Joint J 2013; 95-B(9): 1172–1177. 23997127 10.1302/0301-620X.95B9.31711

[18] Carrothers AD Gilbert RE Jaiswal A et al Birmingham hip resurfacing: the prevalence of failure. J Bone Joint Surg Br 2010; 92(10): 1344–1350. 20884969 10.1302/0301-620X.92B10.23504

[19] Nam D Nunley RM Ruh EL et al Short-term results of birmingham hip resurfacing in the United States. Orthopedics 2015; 38(8): e715–e721. 26270759 10.3928/01477447-20150804-60

[20] Davis ET Olsen M Zdero R et al Femoral neck fracture following hip resurfacing: the effect of alignment of the femoral component. J Bone Joint Surg Br 2008; 90(11): 1522–1527. 18978277 10.1302/0301-620X.90B11.20068

[21] Olsen M Davis ET Whyne CM et al The biomechanical consequence of insufficient femoral component lateralization and exposed cancellous bone in hip resurfacing arthroplasty. J Biomech Eng 2010; 132(8): 081011. 20670060 10.1115/1.4001159

[22] Campbell P Beaule PE Ebramzadeh E et al The John Charnley award: a study of implant failure in metal-on-metal surface arthroplasties. Clin Orthop Relat Res 2006; 453: 35–46. 16906115 10.1097/01.blo.0000238777.34939.82

[23] De Haan R Pattyn C Gill HS et al Correlation between inclination of the acetabular component and metal ion levels in metal-on-metal hip resurfacing replacement. J Bone Joint Surg Br 2008; 90(10): 1291–1297. 18827237 10.1302/0301-620X.90B10.20533

[24] Ollivere B Darrah C Barker T et al Early clinical failure of the Birmingham metal-on-metal hip resurfacing is associated with metallosis and soft-tissue necrosis. J Bone Joint Surg Br 2009; 91(8): 1025–1030. 19651828 10.1302/0301-620X.91B8.21701

[25] Upadhyay A York S Macaulay W et al Medical malpractice in hip and knee arthroplasty. J Arthroplasty 2007; 22(6): 2–7. 10.1016/j.arth.2007.05.003 17823005

